# Metoprolol vs. Diltiazem in Patients with Angina and Non-Obstructive Coronary Artery Disease with or Without Evidence of Coronary Microvascular Spasm on Acetylcholine Testing

**DOI:** 10.3390/jcm14217635

**Published:** 2025-10-28

**Authors:** Angelo Giuseppe Marino, Nello Cambise, Fabio De Benedetto, Ludovica Lenci, Sara Pontecorvo, Federico Di Perna, Giacomo Buonamassa, Antonietta Belmusto, Saverio Tremamunno, Antonio De Vita, Rosangela Capasso, Rocco Antonio Montone, Gaetano Antonio Lanza

**Affiliations:** 1 Dipartimento di Scienze Cardiovascolari e del Torace, Università Cattolica del Sacro Cuore, 00168 Rome, Italy; angelomarino1995@gmail.com (A.G.M.); fabiodebenedetto95@gmail.com (F.D.B.); ludovicalenci@gmail.com (L.L.); sara.pontecorvo15@gmail.com (S.P.); federicodiperna1@gmail.com (F.D.P.); giacomobuonamassa@gmail.com (G.B.); antonietta.belmusto@gmail.com (A.B.); 2 Fondazione Policlinico Universitario A. Gemelli IRCCS, 00168 Rome, Italy; nellocambise@gmail.com (N.C.); tremamunnosaverio@gmail.com (S.T.); antonio.devita90@gmail.com (A.D.V.); roccoantonio.montone@policlinicogemelli.it (R.A.M.); 3 Dipartimento di Medicina Clinica e Molecolare, Divisione di Cardiologia, Sapienza Università di Roma, 00185 Rome, Italy; capassorosangela@gmail.com

**Keywords:** angina syndromes, non-obstructive coronary artery disease, exercise stress test, acetylcholine test, beta blockers, calcium-channel blockers

## Abstract

**Background:** Pharmacologic therapy guided by invasive coronary function tests (CFTs) may improve symptomatic outcomes in patients with angina and non-obstructive coronary artery disease (ANOCA). In this study, we specifically aimed to investigate whether the induction of coronary microvascular spasm (CMVS) by the acetylcholine (Ach) test predicts a better therapeutic effect of calcium-channel blocker therapy compared to beta-blocker therapy. **Methods:** We enrolled 31 ANOCA patients, who were divided into two groups according to the result of Ach testing: 16 patients with CMVS (CMVS group) and 15 patients with a negative test (NEG group). Patients with Ach-induced epicardial spasm were excluded. In an open-label crossover trial, patients were randomly assigned to each receive, for a period of 4 weeks, either metoprolol (50 mg twice daily) or diltiazem (120 mg twice daily). At the end of each 4-week period, patients underwent an ECG–exercise stress test (EST) and were invited to fill out the Seattle Angina Questionnaire (SAQ). **Results:** No significant differences were found between metoprolol and diltiazem in terms of SAQ scores, and ECG-EST results were also largely comparable with the two drug treatments, both in the CMVS group and the NEG group. In particular, the SAQ summary score was 63.1 ± 24 and 66.0 ± 25 (*p* = 0.59) for metoprolol and diltiazem, respectively, in the CMVS group, and 70.9 ± 17 and 74.3 ± 16 (*p* = 0.37) with the two drugs, respectively, in the NEG group. **Conclusions:** Our small open-label study shows that patients with ANOCA with negative Ach test or Ach-induced CMVS show largely comparable short-term symptomatic outcomes and ECG-EST results when treated with either metoprolol or diltiazem.

## 1. Introduction

Up to one-half of patients who undergo elective coronary angiography because of chest pain and about 15% of patients admitted to the hospital with a clinical diagnosis of non-ST-segment elevation acute coronary syndrome (NSTE-ACS) are found to have non-obstructive coronary artery disease [1,2]. In most of these patients, chest pain is caused by myocardial ischemia, which can be induced by different mechanisms, including epicardial coronary artery spasm (CAS), coronary microvascular spasm (CMVS), and impaired coronary microvascular dilatation [3,4].

Invasive coronary functional tests (CFTs) have been recently proposed to identify the mechanism(s) responsible for angina symptoms in patients with non-obstructive coronary artery disease. Although several types of tests can be performed to assess coronary function, two types of tests are usually performed, i.e., the acetylcholine (Ach) and the adenosine tests [5,6]. An Ach test is performed to identify the presence of abnormal epicardial or microvascular vasoconstrictive activity, whereas the adenosine test assesses coronary flow reserve (CFR), a measure of coronary microvascular dilation in the absence of epicardial flow-limiting stenosis [3,4,5,6].

As suggested by the results of the CorMiCa trial [7,8], identifying the mechanism(s) responsible for angina symptoms through CFTs may better guide pharmacologic treatment of patients, resulting in improved symptom control. Recent studies, however, have questioned whether CFTs lead to a significantly better symptomatic outcome in these patients [9,10].

Indeed, it is not completely clear whether a constrictor response to Ach can always identify the true mechanism responsible for angina symptoms in an individual patient, or whether it may simply represent a non-specific vasoreactivity response in at least some patients, in whom other mechanisms are actually responsible for chest pain [3]. Moreover, it remains unclear whether vasodilator therapy with calcium-channel blockers (CCBs) is as effective for CMVS as it is for epicardial CAS and more effective than beta blockers (BBs) in patients with evidence of CMVS during CFTs.

The specific aim of this study was to assess whether in patients with angina and non-obstructive coronary artery disease (ANOCA), who show evidence of CMVS, differences exist in symptomatic outcomes and ECG–exercise stress test (EST) results between treatment with BBs and CCBs. It should be emphasized that this was not a study designed to demonstrate the efficacy of these two classes of anti-ischemic drugs in ANOCA patients, but rather to evaluate whether, in daily clinical practice, prescription at discharge of CCBs to this patient population is actually associated with better outcomes compared to BB therapy. Notably, this is the first clinical trial aimed at comparing a BB and a CCB in this specific subset of patients. Previous studies involving BBs, CCBs, or both have, in fact, only included patients with non-specific endotypes of ANOCA (Table 1) [11,12,13,14,15,16,17]. The outcomes of CMVS patients in this study were compared with those observed in a group of ANOCA patients who had a negative acetylcholine test.

## 2. Methods

### 2.1. Patients

Patients who fulfilled the following inclusion criteria were considered eligible for this study: (1) coronary angiography performed at our hospital because of either episodes of stable, exercise-induced chest pain compatible with typical angina or a clinical picture of unstable angina (i.e., admission to our emergency department for one or more episodes of chest pain at rest, lasting less than 20 min and associated with ischemia-like ST-segment and/or T-wave changes, but without any increase in serum high-sensitivity troponin levels on serial measurements); (2) absence of significant coronary stenosis, defined as >50% reduction in epicardial lumen diameter and/or fractional flow reserve <0.80, assessed during invasive coronary angiography; (3) an Ach test performed during coronary angiography that was negative for the induction of epicardial spasm; (4) no condition that could preclude a symptom-limited EST; (5) no ECG abnormalities that could limit ST-segment analysis during ECG-EST; (6) no evidence of any previous heart disease; (7) no contraindications to the use of either diltiazem or metoprolol, or both; and (8) no serious comorbidities.

Patients were excluded from the study if they had (1) any condition that could preclude a symptom-limited EST; (2) ECG abnormalities that could make ST-segment changes during ECG-EST unreliable (e.g., non-sinus rhythm, conduction disorders, significant basal ST-segment or T-wave changes); (3) evidence of any significant heart disease (valvular, congenital, etc.) or serious comorbidities (e.g., malignancy, acute or chronic inflammatory disease, severe renal or hepatic failure); (4) contraindications to the use of diltiazem, metoprolol, or both (e.g., atrioventricular block, bradycardia, hypotension, asthma, etc.).

### 2.2. Acetylcholine Test

The Ach test was performed during invasive coronary angiography according to a previously described protocol [18]. Briefly, intracoronary infusion of Ach into the left coronary artery was administered at increasing doses of 20, 50, and 100 μg, each over a period of 3 minutes, and with a 3-minute interval between doses. Coronary angiography was performed at the end of each dose, or immediately in the event of chest pain and/or ischemic ECG changes. The test was interrupted in the presence of positive findings or the occurrence of any side effect. If the test result was negative in the left coronary artery, the test was also performed in the right coronary artery by administering Ach doses of 20 and 50 µg only.

To minimize potential confounding from borderline cases, patients were excluded from the study if they showed a focal or diffuse narrowing of the coronary diameter ≥ 75%, accompanied by typical symptoms and/or ischemic ECG changes [19]. CMVS was diagnosed when both typical symptoms and ischemic ECG changes were induced in the absence of epicardial CAS, as defined above.

Based on the result of the Ach test, enrolled patients were divided into two subgroups: patients with CMVS induction (CMVS group) and patients with a negative test result (NEG group).

### 2.3. Study Design

**Figure 1:**
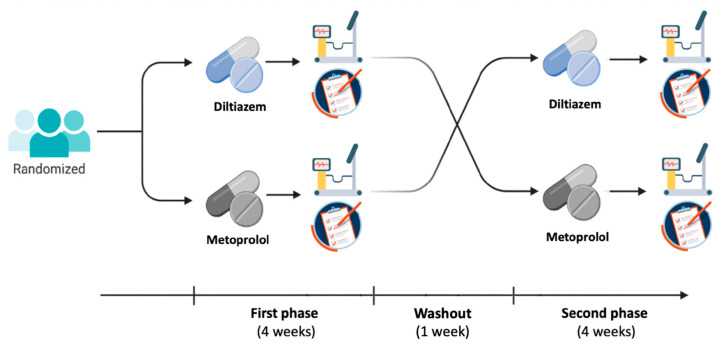
Design of the study.

The study was designed as a prospective, randomized, open-label, crossover clinical trial (Figure 1). Clinical characteristics and cardiovascular risk factors were collected from all patients, who provided their written informed consent to participate in the study, which was approved by the Ethics Committee of Fondazione Policlinico Universitario A. Gemelli IRCCS, Università Cattolica del Sacro Cuore, Rome, Italy (ID: 5404/2023).

**Figure 2:**
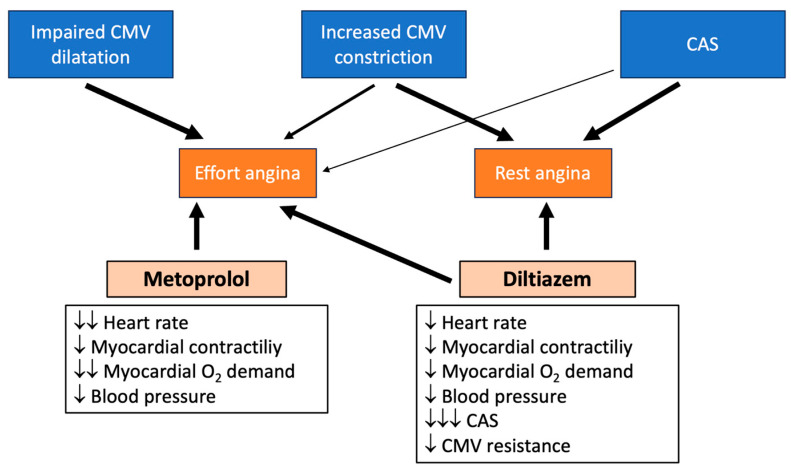
Coronary functional mechanisms involved in ANOCA and mechanisms of action and expected effects of metoprolol and diltiazem (the thickness of arrows indicates the entity of the effect).

All patients were randomized to receive, for a period of 4 weeks, either a cardio-selective BB (metoprolol tartrate 50 mg twice daily) or a non-dihydropyridine CCB (diltiazem 120 mg twice daily). Randomization was performed using a computer-generated sequence of random numbers. At the end of the 4-week period, patients were asked to complete the Seattle Angina Questionnaire (SAQ) and undergo an ECG-EST. After a short 7-day washout period, patients were then switched to the other drug for a new period of 4 weeks, at the end of which they again completed the SAQ and underwent ECG-EST. The main pharmacologic actions of metoprolol and diltiazem and their expected effects on the various endotypes characterizing patients with ANOCA are summarized in Figure 2.

No other anti-ischemic drugs were allowed throughout the study, while all other medications were kept constant. The adherence to therapy was assessed by pill count at the end of each phase and was consistently found to be higher than 95%. The primary endpoint of the study was the angina status, as assessed by the SAQ. As a secondary endpoint, we evaluated the effect of treatments on the ECG-EST results.

#### 2.3.1. Seattle Angina Questionnaire

SAQ is a 19-item self-administered, disease-specific patient-reported outcome with 5 domains: physical limitation; anginal stability; anginal frequency; treatment satisfaction; and disease perception/quality of life [20].

All SAQ domain scores range from 0 to 100 with higher scores indicating less angina, fewer functional limitations, and better quality of life. A SAQ summary score (SAQSS), which provides a global metric of angina severity, is calculated as the average of the domains of angina limitation, angina frequency, and quality of life [21].

#### 2.3.2. Exercise Stress Test

ECG-ESTs were performed without changes to the current patients’ pharmacologic therapy, except for the study drugs. The tests were conducted in the early afternoon, following the standard treadmill Bruce protocol. Three ECG leads (V2, aVF, and V5) were continuously monitored during the test. A standard 12-lead ECG was recorded, and blood pressure was measured using a cuff sphygmomanometer at the onset of the test, at the end of each stage and at peak exercise, as well as at 1 mm ST-segment depression (STD), when angina occurred, and whenever clinically indicated. Multiple 12-lead ECG strips were recorded when STD began to appear to precisely determine the timing of 1 mm STD occurrence.

The test was stopped when one or more of the following endpoints were reached: (1) physical exhaustion; (2) progressive angina (Borg scale > 6); (3) STD > 4 mm; and (4) occurrence of potentially harmful clinical conditions, such as hypotension, severe arrhythmias, or worsening dyspnea.

The test was considered positive for myocardial ischemia when a horizontal or down-sloping STD of >1 mm was observed in at least one lead. The ECG strips of both tests were evaluated blindly and independently by two expert cardiologists. Discrepancies were resolved by consensus under the supervision of a third expert cardiologist.

For each ECG-EST, we recorded heart rate (HR), systolic and diastolic blood pressure (BP) at baseline, at 1 mm STD, and at peak exercise, as well as time to 1 mm STD and peak exercise. Maximal STD was also recorded. For statistical analyses, the values at peak exercise were used for those at 1 mm STD when STD did not occur during the test.

### 2.4. Statistics

Based on data from previous studies [7,8,22,23], to achieve a statistical power of 80% and a significance level of *p* < 0.05 for detecting a mean difference > 15 points (with a standard deviation of 19) in any SAQ domain between the two treatments, a sample size of 14 patients per group was required. The normal distribution of data was assessed using the Kolmogorov–Smirnov test. Continuous variables are reported as means ± standard deviations and were compared using paired *t*-test. Categorical variables are reported as proportions and were compared using Fisher’s exact test. Positivity of ECG-EST and EST-induced angina were compared using the McNemar test. A *p* < 0.05 was required for statistical significance. The SPSS 28.0 statistical software (SPS Inc., Florence, Italy) was used for the analysis of data. Data from this study are available upon request to the corresponding author.

## 3. Results

Overall, 31 patients were considered eligible for the study: 16 patients (52%) with induction of CMVS (CMVS group) and 15 patients (48%) with a negative Ach test (NEG group). The main findings of the Ach test are summarized in Table 2. Three patients (two in CMVS group and one in NEG group) were lost to follow-up and were subsequently excluded. Therefore, 28 patients concluded both phases of the study: 14 (50%) in the CMVS group and 14 (50%) in the NEG group. The main clinical characteristics of the patients are summarized in Table 3.

### 3.1. Angina Status

The main results of symptomatic outcomes are summarized in Table 4. As shown, there were no significant differences in any of the SAQ domains between treatment with metoprolol and diltiazem, both in patients from the CMVS group and the NEG group.

Patients with CMVS reported a slightly higher quality of life score during treatment with diltiazem, but the difference did not reach statistical significance (*p* = 0.08).

**Figure 3:**
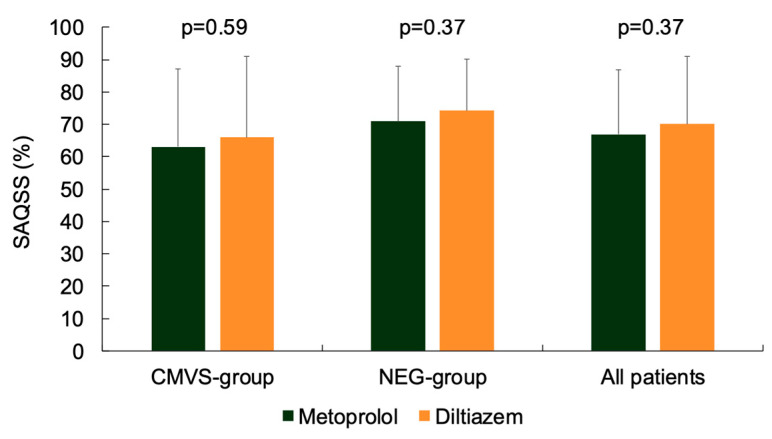
SAQSS after treatment with metoprolol or diltiazem in the CMVS group and in the NEG group.

SAQSS was 63.1 ± 24 and 66.0 ± 25 (*p* = 0.59) with metoprolol and diltiazem, respectively, in the CMVS group, and 70.9 ± 17 and 74.3 ± 16 (*p* = 0.37) with the two drugs, respectively, in the NEG group (Figure 3).

No serious side effects were reported with either type of drug (Table 5). Subgroup analyses of patients with various cardiovascular risk factors and types of angina (on exertion or at rest) did not reveal any significant differences in angina status between the two types of treatment (Supplementary Table S2). Similarly, the comparison of SAQ data obtained at the end of the first phase of the cross-over study also did not reveal any significant difference between the patients taking metoprolol and those taking diltiazem (Supplementary Table S3).

### 3.2. Exercise Stress Test

Six patients refused to undergo the second ECG-EST (four in the CMVS group and two in the NEG group) and were therefore excluded from the ECG-EST data analyses. The clinical characteristics of these patients did not differ significantly from those who completed the ECG-EST protocol (Supplementary Table S1). The main ECG-EST results of patients who completed both treatment phases are summarized in Table 6.

In the CMVS group (n = 10), ECG-EST was positive for myocardial ischemia in 7 patients (70%) with metoprolol and in 8 patients (80%) with diltiazem. In the NEG group (n = 12), the ECG-EST was positive in 6 patients (50%) with metoprolol and 7 patients (58%) with diltiazem.

There were no major clinically relevant differences in most ECG-EST parameters between metoprolol and diltiazem, although in the NEG group, HR at 1 mm ST-segment depression was higher (*p* = 0.04) and ECG-EST duration was longer (*p* = 0.01) with diltiazem compared with metoprolol.

## 4. Discussion

The most relevant finding of the present study is that patients with ANOCA, with either Ach-induced CMVS or a negative Ach test, exhibited large comparable symptomatic outcomes and ECG-EST results when treated with metoprolol or diltiazem, suggesting that the induction of CMVS by Ach testing, as established by current diagnostic methods, does not predict a significant better response to treatment with the vasodilator CCB. Although the SAQ quality-of-life domain tended to be higher (*p* = 0.08) and time to 1 mm STD was lower (*p* = 0.04) with diltiazem than metoprolol in the CMVS group, overall, no major differences appeared to be present between the two treatments.

Previous studies have attempted to determine whether BBs and CCBs differ in relieving symptoms or improving ischemic ECG abnormalities in patients with microvascular angina, but conflicting results have been reported [14,15,16,17,24]. A possible reason for these discordant findings is that previous studies were conducted on populations with heterogeneous mechanisms underlying their angina symptoms. The increasing use of invasive CFTs in patients with ANOCA has recently made it possible to prescribe drug therapy tailored to individual patients based on the abnormal coronary vasomotor responses detected by these tests, mainly including adenosine and Ach tests [25,26].

The CorMiCA trial demonstrated that pharmacologic therapy guided by the results of CFTs significantly improved angina status of this patient population compared to empirical therapy, with a difference of 11.68 points in the change of the SAQ summary score after 6-month follow-up [7]. Notably, the CorMiCA trial showed that patients with evidence of epicardial spasm benefited from a greater use of vasodilator drugs (CCBs and nitrates), whereas patients with coronary microvascular dysfunction benefited more from a greater use of BBs, inhibitors of the angiotensin system and statins [8]. More recently, similar results were reported in the ILIAS-ANOCA trial, which reported a significant difference of 9.4 points in the change of the SAQ summary score after 6 months of therapy in ANOCA patients treated with CFT-guided treatment versus empirical treatment [27].

However, some other studies failed to confirm these results, showing no significant improvement with CFT-guided therapy compared to empirical therapy [9,10]. The EDIT-CMD trial, in particular, did not demonstrate significant improvement of symptoms with diltiazem in ANOCA patients with evidence of epicardial or coronary microvascular spasm at CFTs, which appeared to be mainly related to a lack of effects of diltiazem on CMVS [10].

Our study provides further insights on this important aspect of management of ANOCA patients. Contrary to expectations, we found no significant differences in the symptomatic outcome, and only minor differences in ECG-EST results, between treatment with either metoprolol or diltiazem, regardless of the CMVS induction during the Ach test. This result suggests either that CMVS was not the true mechanism underlying clinical angina symptoms in the CMVS group or that CCBs are not more effective than BBs in this patient population. The latter possibility is supported by the evidence that, in patients with demonstrated CMVS, the recurrence or persistence of angina episodes was already reported to be high despite CCB therapy [28,29], which has recently been confirmed by the EDIT-CMD trial (Efficacy of Diltiazem to Improve Coronary Microvascular Dysfunction: A Randomized Clinical Trial) [10]. In this trial, 85 patients with ANOCA and positive CFTs were randomized to receive diltiazem or placebo. CFTs were repeated after 6 weeks in 73 patients (38 diltiazem, 35 placebo). No significant improvement in CFT results was observed with diltiazem versus placebo; interestingly, however, diltiazem was associated with a significant reduction in Ach-induced epicardial spasm (from 50% to 32%), but also with a concomitant significant increase in CMVS (from 21% to 37%), whereas no changes were observed in patients receiving placebo. These results were associated with lack of symptomatic benefits from diltiazem therapy [28,29,30,31]. These findings confirm previous observations that Ach-induced CAS may coexist with, and obscure, Ach-induced CMVS in at least a subset of ANOCA patients [30]. Furthermore, they suggest that CMVS, rather than CAS, can be responsible for angina symptoms in most of the latter patients, which could explain the lower efficacy of CCB therapy in controlling symptoms. Previous studies, indeed, suggested that CCBs may have limited effects on CMVS compared with CAS [28,29,30,31].

Notably, these previous studies and our study underscore the need to consider the results of CFTs with caution, as they may not identify the true, or even the sole, mechanism in individual patients with ANOCA.

Importantly, whereas BBs should possibly be avoided in patients with CAS [24,32], no study has demonstrated that the presence of CMVS predicts a worse symptomatic outcome in patients treated with BBs. In contrast, BBs are currently used safely and effectively as first-line therapy in patients with ANOCA without any clinical evidence of vasospastic variant angina [33,34], which may include a variable proportion of patients in whom CMVS is the mechanism responsible for symptoms [35,36].

Finally, it is worth noting that similar effects of metoprolol and diltiazem were also observed in patients who had a negative Ach test. Although we did not assess coronary microvascular dilator function in these patients, the comparable symptomatic outcomes with the two types of drugs may suggest that they may also have largely similar effects in patients with impaired coronary microvascular dilator function that are known to constitute a large proportion of this group of patients [3,4,5,6].

### Limitations of the Study

Some limitations of our study should be acknowledged. First, this was a single-center study with a small sample size, which was powered to detect only relatively large differences in angina status (≥15 points in any SAQ item) between the two treatment groups and was therefore underpowered (type II error) to detect smaller but potentially significant differences; thus, whether smaller significant differences actually exist between BB and CCB therapy in our patient population should be addressed in larger studies, although such differences may be of limited clinical relevance.

Second, this is an open-label study, and therefore the possibility of biases due to the lack of blinding cannot be excluded; accordingly, double-blind trials are warranted to validate our data.

Third, we did not perform a basal assessment of SAQ and ECG-EST after a run-in placebo phase; therefore, we cannot establish whether, and to what extent, drug therapy had a favorable impact on clinical outcome of patients. However, in this study, we were specifically interested in assessing whether the routine prescription of CCBs or BBs in ANOCA patients with CVMS at Ach testing would result in differences in symptomatic outcome, rather than evaluating the therapeutic effects of the two drugs, which have already been established in patients with angina and normal coronary arteries [14,15,16,17,24,37].

Fourth, the effects of drugs were assessed after a short period of therapy (4 weeks); therefore, further studies should clarify whether different effects might emerge over long-term therapy.

Fifth, several patients were concomitantly taking therapies that could have variably influenced the effects of the study drugs through variable actions on microvascular function, endothelial function, inflammation, electrolyte balance, and platelet activity, thus possibly masking differences in the therapeutic effects of the two drugs.

Finally, CFR was not assessed in our patients and, therefore, we cannot establish the presence of a concomitant impairment of coronary microvascular dilation.

## 5. Conclusions

Our data show that patients with ANOCA, whether they have evidence of CMVS or a negative response to the Ach test, exhibit largely comparable symptomatic outcomes and ECG-EST results at short-term follow-up when treated with either metoprolol or diltiazem, thus suggesting that these two types of drugs could be prescribed interchangeably a first-line therapy in these subsets of patients in routine clinical practice.

Since our data were obtained in a small, open-label trial, they should be verified in larger studies with longer duration of treatment. Furthermore, future research should clarify whether combination therapy (BB/CCB) could lead to significant improvement in the clinical efficacy of treatment and whether other classes of drugs may contribute significantly to improving angina status and quality of life in the specific group of patients with Ach-induced CMVS.

## Figures and Tables

**Table 1:** Overview of previous studies evaluating BB and CCB therapy in patients with ANOCA.

	Study Design	Population	No. pts	Drugs Tested	Outcome	Results
Lanza [11]	Crossover, randomized, double-blind	Effort angina Positive ECG-EST NCAs	10	Atenolol vs. amlodipine vs. ISMN 5-mononitrate	Angina episodes	Only atenolol significantly reduced angina episodes
Romeo [12]	Crossover, double-blind, randomized	Effort angina Positive ECG-EST NCAs No CAS	30	Acebutolol vs. verapamil	ECG-EST	Verapamil improved ECG-EST in the whole population; acebutolol improved ECG-EST in patients with enhanced sympathetic activity only
Ferrini [13]	Crossover, randomized, single-blind	Effort angina Positive ECG-EST NCAs No CAS	13	Propanolol vs. diltiazem vs. placebo	ECG-EST	Diltiazem, but not propranolol, improved ECG-EST results
Leonardo [14]	Crossover, randomized, double-blind	Effort angina Positive EST NCAs	16	Atenolol vs. trimetazidine vs. placebo	Angina episodes, ECG-EST, echocardiograhy	Atenolol, but not trimetazidine, reduced angina episodes, ECG-EST abnormalities, and improved diastolic function
Cannon [15]	Crossover, double-blind, randomized	Angina NCAs Abnormal vasodilator reserve	26	Verapamil vs. nifedipine vs. placebo	Angina episodes, nitrate use, ECG-EST	CCBC reduced angina episodes and nitrate consumption and improved ECG-EST results.
Ozcelik [16]	Crossover, non-randomized open-label,	Effort angina Positive ECG-EST NCAs No CAS	18	Nisoldipine vs. ramipril	Angina episodes, nitrate use, ECG-EST	Nisoldipine and ramipril reduced angina episodes and nitrate consumption and improved ECG-EST results.
Li [17]	Randomized, double-blind trial	Angina NCAs Coronary slow flow	80	Diltiazem vs. placebo	Angina episodes, ECG-EST, CBF	Diltiazem reduced angina episodes and improved ECG-EST results, and coronary blood flow

BB = beta blocker; CAS = coronary artery spasm; CBF = coronary blood flow; CCB = calcium-channel blocker; ECG-EST = electrocardiogram–exercise stress test; ISMN = isosorbide-5-mononitrate; NCAs = normal coronary arteries.

**Table 2:** Detailed intracoronary acetylcholine testing results.

	CMVS Group (n = 16)	NEG Group (n = 15)	*p*
Angina	16 (100%)	5 (33%)	<0.001
Ischemic ECG changes	16 (100%)	1 (7%)	<0.001
Mild focal vasoconstriction (<75%)	3 (19%)	1 (7%)	0.32
Mild diffuse vasoconstriction (<75%)	2 (13%)	3 (20%)	0.57
Ach maximal dose	55 ± 23	100 ± 0	<0.001

CMVS = coronary microvascular spasm; ECG = electrocardiogram; Ach = acetylcholine.

**Table 3:** Main clinical characteristics of the two groups of patients.

	CMVS Group (n = 16)	NEG Group (n = 15)	*p*
Age (years)	56 ± 10	62 ± 13	0.15
Sex (M/F)	3/13	5/10	0.43
*Cardiovascular risk factors*			
Family history of CVD	4 (25%)	6 (40%)	0.46
Hypertension	6 (38%)	9 (60%)	0.29
Active smoking	5 (31%)	3 (20%)	0.69
Hypercholesterolemia	6 (38%)	8 (53%)	0.48
Diabetes	3 (19%)	3 (20%)	0.93
*Clinical presentation*			
Stable angina	5 (31%)	8 (53%)	0.78
Unstable angina	11 (69%)	7 (47%)	0.29
*Other cardiovascular drugs*			
ACE-inhibitors/ARBs	7 (44%)	7 (47%)	0.87
Diuretics	1 (6%)	2 (13%)	0.60
Statins	7 (44%)	4 (27%)	0.46
Aspirin	10 (63%)	8 (53%)	0.72

ACE = angiotensin-converting enzyme; ARB = angiotensin receptor blockers; CVD = cardiovascular disease.

**Table 4:** Results of Seattle Angina Questionnaire after treatment with metoprolol or diltiazem.

	CMVS Group (n = 14)			NEG Group (n = 14)		
	Metoprolol	Diltiazem	*p*	Metoprolol	Diltiazem	*p*
Physical limitation	70.7 ± 31	67.9 ± 36	0.79	77.9 ± 19	80.5 ± 17	0.58
Angina stability	59.4 ± 37	61.9 ± 35	0.74	53.6 ± 35	71.1 ± 32	0.14
Angina frequency	67.5 ± 30	70.4 ± 27	0.47	77.9 ± 17	80.7 ± 15	0.45
Treatment satisfaction	71.9 ± 22	73.6 ± 26	0.68	72.8 ± 21	77.6 ± 19	0.26
Quality of life	51.1 ± 27	59.7 ± 28	0.08	57.1 ± 24	61.5 ± 28	0.47

**Table 5:** Summary of side effects observed during the two treatment phases of the study.

	CMVS Group (n = 14)	NEG Group (n = 14)	*p*
Metoprolol			
Hypotension	0 (0%)	1 (7%)	0.31
Fatigue	3 (21%)	2 (14%)	0.62
Dizziness	1 (7%)	1 (7%)	-
Diltiazem			
Hypotension	2 (14%)	1 (7%)	0.34
Headache	2 (14%)	3 (21%)	0.62
Constipation	1 (7%)	0 (0%)	0.31

**Table 6:** Results of ECG-EST after treatment with metoprolol or diltiazem.

	CMVS Group (n = 10)			NEG Group (n = 12)		
	Metoprolol	Diltiazem	*p*	Metoprolol	Diltiazem	*p*
*Rest*						
HR (bpm)	70 ± 21	73 ± 9	0.57	70 ± 10	74 ± 10	0.19
Systolic BP (mmHg)	129 ± 18	121 ± 16	0.57	127 ± 14	131 ± 15	0.42
Diastolic BP (mmHg)	75 ± 9	73 ± 8	0.24	78 ± 6	78 ± 9	0.73
*1 mm STD*						
HR (bpm)	119 ± 19	125 ± 17	0.04	122 ± 24	130 ± 24	0.12
Systolic BP (mmHg)	154 ± 17	146 ± 13	0.07	144 ± 17	155 ± 14	0.04
Diastolic BP (mmHg)	81 ± 8	75 ± 7	0.06	83 ± 11	86 ± 9	0.27
Time to 1 mm (s)	424 ± 192	399 ± 173	0.55	401 ± 149	420 ± 172	0.53
*Peak exercise*						
HR (bpm)	129 ± 15	137 ± 13	0.07	132 ± 21	139 ± 21	0.07
Systolic BP (mmHg)	168 ± 13	155 ± 18	<0.01	158 ± 25	161 ± 19	0.62
Diastolic BP (mmHg)	85 ± 10	79 ± 6	0.07	87 ± 10	87 ± 9	0.90
EST duration (s)	508 ± 137	491 ± 118	0.44	468 ± 96	514 ± 99	0.01
Positive EST	7 (70%)	8 (80%)	1.0	6 (50%)	7 (58%)	1.0
Angor	1 (10%)	1 (10%)	1.0	6 (50%)	6 (50%)	1.0
Maximal STD (mm)	1.1 ± 0.8	1.1 ± 0.7	0.78	1.0 ± 1.2	1.0 ± 1.1	0.75

BP = blood pressure; HR = heart rate; STD = ST depression; EST = exercise stress test.

## Data Availability

The data presented in this study are available upon reasonable request from the corresponding author.

## Supplementary Materials

The following supporting information can be downloaded at https://www.mdpi.com/article/10.3390/jcm14217635/s1, Table S1: Baseline clinical characteristics of patients who completed vs those who did not complete the ECG-EST protocol. Table S2: Subgroup analyses of SAQSS across major clinical characteristics subgroups in all patients. Table S3: Results of sensitivity analyses comparing SAQ results of the first treatment phase only. Table S4: Correlation between ECG-EST parameters and SAQSS in all patients.

## Abbreviations

The following abbreviations are used in this manuscript:

Ach	Acetylcholine
ANOCA	Angina with non-obstructive coronary artery disease
BBs	Betablockers
CAS	Coronary artery spasm
CCBs	Calcium-channel blockers
CFR	Coronary flow reserve
CFTs	Coronary function tests
CMVS	Coronary microvascular spasm
ECG-EST	ECG–exercise stress test
SAQ	Seattle Angina Questionnaire
STD	ST-segment depression
