# Supplementary material for: Metoprolol vs. Diltiazem in Patients with Angina and Non-Obstructive Coronary Artery Disease with or Without Evidence of Coronary Microvascular Spasm on Acetylcholine Testing
Source: J Clin Med. 2025 Oct 28;14(21):7635. doi: 10.3390/jcm14217635 (PMC12608405; doi:10.3390/jcm14217635)
Supplement: Supplementary file 1 [file jcm-14-07635-s001.zip › jcm-3882175-supplementary.pdf]

SUPPLEMENTARY TABLE

Supplementary Table S1. Baseline clinical characteristics of patients who completed vs those who did not complete the ECG-EST protocol.

	**Patients with two ECG-ESTs (n=22)**	**Patients with one ECG-EST (n=6)**	***p***
Age (years)	59±12	58±12	0.86
Sex (M/F)	8/14	0/6	0.08
Cardiovascular risk factors			
Family history of CVD	7 (32%)	2 (33%)	0.94
Hypertension	12 (55%)	3 (50%)	0.84
Active smoking	7 (32%)	1 (17%)	0.47
Hypercholesterolemia	12 (55%)	2 (33%)	0.36
Diabetes	5 (23%)	0 (0%)	0.19
Clinical presentation			
Stable angina	9 (41%)	2 (33%)	0.74
Unstable angina	12 (59%)	4 (66%)	0.59
Other cardiovascular drugs			
ACE-inhibitors/ARBs	13 (59%)	1 (17%)	0.07
Diuretics	2 (10%)	1 (17%)	0.59
Statins	9 (41%)	2 (33%)	0.74
Aspirin	12 (55%)	4 (67%)	0.59

ACE=angiotensin-converting enzyme; ARB=angiotensin receptor blockers; CVD=cardiovascular disease

Supplementary Table S2. Subgroup Analyses of SAQSS across major clinical characteristics subgroups in all patients.

	Metoprolol	Diltiazem	*p*
Hypertensive (n=15)	68±21	73±17	0.19
Not hypertensive (n=13)	66±22	66±26	0.96
Men (n=8)	76±10	79±12	0.57
Women (n=20)	64±23	67±24	0.43
Hypercholesterolemic (n=14)	68±22	71±21	0.57
Non hypercholesterolemic (n=14)	66±20	69±23	0.38
Smokers (n=8)	66±17	62±19	0.51
Nonsmokers (n=20)	67±23	73±22	0.12
Diabetics (n=5)	76±16	76±17	0.97
Not diabetics (n=23)	65±22	69±23	0.32
Patients with effort angina (n=11)	63±17	71±17	0.17
Patients with angina at rest (n=17)	69±23	69±25	0.99

Supplementary Table S3. Results of sensitivity analyses comparing SAQ results of the first treatment phase only.

	Metoprolol (n=14)	Diltiazem (n=14)	*p*
Angina stability	64±36	66±37	0.90
Physical limitation	82±22	67±30	0.12
Angina frequency	77±25	76±18	0.93
Quality of life	63±25	55±30	0.47
Treatment satisfaction	77±19	75±20	0.86
SAQSS	74±21	66±21	0.31

SAQSS=Seattle Angina Questionnaire summary score

Supplementary Table S4. Correlation between ECG-EST parameters and SAQSS in all patients.

	Metoprolol		Diltiazem	
	r	*p*	r	*p*
1 mm STD				
HR	0.27	0.30	0.15	0.60
Systolic BP	0.06	0.81	0.11	0.69
Time to 1 mm	-0.06	0.81	0.07	0.80
Peak				
HR	-0.07	0.74	0.16	0.44
Systolic BP	0.47	**0.02**	-0.19	0.36
EST duration	0.03	0.89	-0.22	0.26
Maximal STD	0.34	0.12	-0.16	0.57

SAQSS=Seattle Angina Questionnaire summary score; HR=heart rate; BP=blood pressure; STD=ST-segment deviation; EST=exercise stress test.
